# Treatment Outcomes and Costs of Providing Antiretroviral Therapy at a Primary Health Clinic versus a Hospital-Based HIV Clinic in South Africa

**DOI:** 10.1371/journal.pone.0168118

**Published:** 2016-12-12

**Authors:** Lawrence C. Long, Sydney B. Rosen, Alana Brennan, Faith Moyo, Celeste Sauls, Denise Evans, Shookdev L. Modi, Ian Sanne, Matthew P. Fox

**Affiliations:** 1 Department of Internal Medical, School of Clinical Medicine, Faculty of Health Sciences, University of Witwatersrand, Johannesburg, South Africa; 2 School of Public Health, Faculty of Health Sciences, University of Witwatersrand, Johannesburg, South Africa; 3 Health Economics and Epidemiology Research Office, Wits Health Consortium, Johannesburg, South Africa; 4 Center for Global Health & Development, Boston University, Boston, Massachusetts, United States of America; 5 Right to Care, Johannesburg, South Africa; 6 Department of Epidemiology, Boston University School of Public Health, Boston, Massachusetts, United States of America; Katholieke Universiteit Leuven Rega Institute for Medical Research, BELGIUM

## Abstract

**Background:**

In 2010 South Africa revised its HIV treatment guidelines to allow the initiation and management of patients on antiretroviral therapy (ART) by nurses, rather than solely doctors, under a program called NIMART (Nurse Initiated and Managed Antiretroviral Therapy). We compared the outcomes and costs of NIMART between the two major public sector HIV treatment delivery models in use in South Africa today, primary health clinics and hospital-based HIV clinics.

**Methods and findings:**

The study was conducted at one hospital-based outpatient HIV clinic and one primary health clinic (PHC) in Gauteng Province. A retrospective cohort of adult patients initiated on ART at the PHC was propensity-score matched to patients initiated at the hospital outpatient clinic. Each patient was assigned a 12-month outcome of alive and in care or died/lost to follow up. Costs were estimated from the provider perspective for the 12 months after ART initiation. The proportion of patients alive and in care at 12 months did not differ between the PHC (76.5%) and the hospital-based site (74.2%). The average annual cost per patient alive and in care at 12 months after ART initiation was significantly lower at the PHC (US$238) than at the hospital outpatient clinic (US$428).

**Conclusions:**

Initiating and managing ART patients at PHCs under NIMART is producing equally good outcomes as hospital-based HIV clinic care at much lower cost. Evolution of hospital-based clinics into referral facilities that serve complicated patients, while investing most program expansion resources into PHCs, may be a preferred strategy for achieving treatment coverage targets.

## Introduction

With over 2.6 million people on HIV treatment, South Africa has had unprecedented success in rolling out its public sector antiretroviral therapy (ART) program[1]. Despite this success, further expansion to 5.7 million patients on treatment by 2018/2019 [2] will be needed for South Africa to reach the “90-90-90” targets established by the World Health Organization [3], and current budgetary resources are not sufficient [2]. Expanding access to ART at the lowest possible cost is therefore essential, for South Africa and for many other countries facing similar challenges.

When the South African national antiretroviral therapy (ART) program began in 2004, it relied primarily on hospital-based HIV clinics with services delivered by doctors [4]. In 2010, in the face of increasing demand for care and limited personnel, South Africa revised its treatment guidelines to allow the initiation and management of patients on ART by nurses at both hospitals and primary health clinics (PHCs) [5, 6] under a program known as Nurse Initiated and Managed Antiretroviral Therapy (NIMART).

NIMART shifted the focus of the South African treatment program from doctors to nurses and from hospitals to PHCs, increasing the number of accredited ART delivery facilities sites from 496 to 4333 [7]. With the advent of NIMART, South Africa now has two major public sector ART delivery models: centralized, hospital-based HIV outpatient clinics, which serve roughly 12% of patients; and decentralized, full service, primary health clinics, which serve about 85% (authors’ data). Both decentralization and task shifting have been shown to generate health outcomes equivalent to or better than centralized, doctor-led care [8–12]. A recent Cochrane review concluded that there was moderate evidence showing that task shifting for HIV management did not decrease the quality of care and, for patients who were initiated on ART by nurses, may reduce patient loss to follow-up [13].

While several studies have examined outcomes of task shifting and decentralization in South Africa [14–24], few have included both ART initiation and management by nurses in routine public sector care without external resources. Neither of the two available cost estimates, moreover, reflect routine NIMART implementation [17, 25]. There is also no evidence of how NIMART fares at different levels of the public health system; for example primary health clinic relative to traditional hospital-based HIV outpatient clinic, under the normal conditions and constraints of a typical public sector setting. We used routinely collected patient data and actual resource utilization and cost records to evaluate the outcomes and costs of NIMART at a hospital outpatient HIV clinic compared to primary health clinic and provide evidence to guide future program expansion.

## Methods

### Study sites

The study was conducted at one hospital-based outpatient HIV treatment clinic and one primary health clinic. Both sites are situated in the same municipality (pop. 360,000) in West Rand District of Gauteng Province, South Africa. The district was one of the early adopters of NIMART across all its facilities. The two sites are seven kilometers apart and serve similar populations.

The hospital-based HIV clinic is a large regional hospital hereafter referred to as the ‘HIV outpatient clinic’. As of March 2014 it had 4,396 patients actively on ART and saw an average of 1,956 ART patients per month. It originally provided ART initiation and management by doctors, with nurse support for screening and monitoring. With the introduction of NIMART, responsibility for these services was progressively shifted to nurses. During the study period, the HIV outpatient clinic had one dedicated, full time doctor and sixteen nurses (including enrolled nurse assistants and managers).

The primary health clinic (PHC), hereafter referred to as “the PHC”, had as of March 2014 1,958 patients actively on ART and saw approximately of 884 HIV patients for ART pickup in March 2014, which was roughly 11% of the patient load. As of January 2012, HIV was fully integrated into general chronic disease care at the site. During the study period (2012) it had fifteen clinical staff (nurses and a doctor) serving all patients seeking care at the clinic for all conditions.

### Sample selection and matching

We constructed a retrospective cohort of adult (≥18 years) patients initiated on ART at the PHC and matched them to patients initiated at the HIV outpatient clinic. To be included in the cohort, patients had to have at least 15 months of potential follow up as of the date of data collection, not have transferred to another site during their first year after initiation, having initiated after 1 January 2011 (NIMART policy in place) and have a complete patient record available for review. Data collection commenced on 15 January 2014 at the PHC and 1 March 2014 at the HIV outpatient clinic, resulting in the cohorts including patients initiated prior to 15 October 2013 and 1 December 2013 at the PHC and HIV outpatient clinic respectively. At the PHC we enrolled the first 260 patients based on date of initiation who met these inclusion criteria. These patients were then matched to hospital outpatient clinic patients 1:3 on gender, age at initiation (18–30, 31–40, 41–50, >50 years), baseline ART regimen, and baseline CD4 count (0–100, 101–200, 201–350, 350 cells/mm^3^). Viral load is not routinely performed at initiation and could therefore not be included as a matching variable. We used propensity score matching without replacement [26] using the Vmatch macro in SAS [27] to identify a comparison population from among all patients in the HIV outpatient clinic database who initiated ART after 1 January 2011 and met the study inclusion criteria.

### Outcomes data and analysis

Clinical data were collected from paper files and the national electronic HIV register (Tier.net) at the primary health clinic and from a separate electronic patient record (TherapyEdge ^™^) at the HIV outpatient clinic. Study data collectors extracted data from the paper files at the primary health clinic and site staff routinely captured the data found in Tier.net and the Therapy Edge datasets. All three of these sources contained only routinely collected data. Fields collected for the first 12 months following ART initiation for each patient included baseline CD4 count, date of ART initiation, resource usage (visits, laboratory tests, drugs), and outcome at 12 months. CD4 count and viral load results up to 15 months were also collected to assign 12-month outcomes. All patient level data were collected using a study specific database designed and managed in CS Pro (United States Census Bureau, 2014).

Each patient was assigned a single primary, 12-month outcome of either “alive and in care” or “died / lost to follow-up (LTFU)”. If a patient died before 12 months or was >3 months late for their last scheduled visit within the 12-month follow up period (lost to follow-up) then the patient was assigned to the outcome “died / LTFU”. All other patients were considered “alive and in care”. Secondary outcomes based on virologic and immunologic status were also assigned. Patients with a viral load ≥ 1,000 copies/mm^3^ between 9–15 months after treatment initiation were classified as “unsuppressed viral load”. Patients with a CD4 count in month 9–15 showing a decrease of >30% from the highest recorded CD4 count between 0–12 months or below the CD4 count at ART initiation were classified as “immunologic failure”. For laboratory results we used the test result reported between 9 and 15 months that was closest to the 12-month endpoint. We then compared the primary and secondary outcomes over the first 12 months of treatment between sites using crude risk ratios with corresponding 95% confidence intervals. Data cleaning, analysis of the patient level database and creation of the resource utilization database were done using SAS software, version 9.3 (SAS Institute, 2012).

Through sensitivity analysis, we investigated the effect of missing paper files excluded at the PHC (no patients were excluded at the HIV outpatient clinic because of missing files). Data on age, gender, baseline CD4 count, and 12-month primary outcome were collected from Tier.net for patients with missing files, where possible. In the sensitivity analysis, all primary health clinic patients were then matched on age, gender and baseline CD4 count to the hospital outpatient clinic and outcomes were compared. The data available on Tier.net were not sufficient to extract resource utilization and calculate costs. The analytic cohort therefore only included patients who had a paper patient file at the primary health clinic.

### Cost data and analysis

Costs were estimated from the provider perspective for the 12 months after ART initiation using standard costing methods, as outlined in Table 1 and described in previous publications [17, 28]. Resources incurring costs included drugs, diagnostics, clinical staff, space, equipment and other shared services (i.e. other staff, utilities, etc.). Variable resource usage was determined from patient records; utilization of fixed resources, such as space and shared services, was estimated from site records. The quantity of each resource used by each patient was then multiplied by the associated unit cost to determine a total cost per patient. All unit costs were in 2014 South African Rand (ZAR) or adjusted to 2014 and reported costs were converted to United States Dollar (USD) at the average exchange rate prevailing during 2014 of 10.83:1 (ZAR:USD). Average resource utilization per patient year is presented by site; average cost per patient is presented by site, cost category, and primary outcome with a 95% confidence interval, where appropriate. The resource utilization and cost analysis were done in Excel 2013 (Microsoft).

**Table 1:** Methods for estimating costs.

Type of cost	Method for estimating cost
***VARIABLE COSTS (RESOURCES RECORDED IN PATIENT MEDICAL RECORDS)***	
*Drugs*, *diagnostics*, *and other services reported in individual subjects’ medical records*	Unit cost obtained from suppliers or site and multiplied by actual resource usage for each patient.
*Staffing (visits)*	The specific staff cadres providing service for each patient visit is not recorded in patient files. A uniform staffing cost is estimated per patient visit at each site based on total staff cost divided by the total visits during the period. The actual number of visits made by the patient is then multiplied by this cost.
***FIXED COSTS (RESOURCES USED FOR CLINIC OPERATION*, *NOT ALLOCATED TO INDIVIDUAL PATIENTS)***	
*Buildings and utilities*	The space of the building was measured and an average rental cost per square metre obtained from the property market for commercial properties in the area was applied. Utilities were not measured at the study sites and so an estimated utility cost per square metre was determined based on a property with basic utility demands (i.e. basic electronic equipment and lighting, air-conditioning, water for cleaning and sanitation).
*Equipment*	All computer and related equipment was inventoried and costed using the appropriate unit costs and expected working life. Basic furnishings and equipment were costed by including a 10% markup on the rental.
*Supplies*	This included all non-drug and non-diagnostic related supplies (i.e. gloves, paper, pens). Supply usage was not well recorded at the facilities. The available information was captured and an estimated cost was calculated per month per patient visit. The same supply cost per visit was applied to both sites in order to prevent the costs being biased towards a site with incomplete supply records.

The study protocol was reviewed by the Institutional Review Boards of the University of Witwatersrand and Boston University, who approved the collection of the data without informed consent and the use of an anonymous analytic dataset.

## Results

### Sample characteristics

Sample selection is outlined in S1 Fig. A sample of 260 patients was selected from the primary health clinic, of whom 60 were excluded (55 missing files, 3 missing baseline CD4 count, 2 transferred within 12 months of initiating). The final analytic data set therefore included 200 patients from the primary health clinic matched with 600 patients from the HIV outpatient clinic. The patients excluded from the PHC sample because of missing data were similar to those included in terms of age and gender but had a slightly higher baseline median (IQR) CD4 count (208 cells/mm^3^ (122–269 cells/mm^3^)) than those included (162 cells/mm^3^ (87–257 cells/mm^3^)).

Characteristics of the sample are described in Table 2. The median age was 34 years (IQR: 28–41 years) and median CD4 count 151 cells/mm^3^ (IQR: 69.5–239.5 cells/mm^3^); two thirds were female. Most patients were initiated on a baseline regimen of lamivudine and tenofovir with either efavirenz (83%) or nevirapine (8%). There were no important differences in sample characteristics between the two sites.

**Table 2:** Baseline characteristics at HIV treatment initiation.

Characteristics	Total	HIV Outpatient Clinic	Primary Health Clinic
	n = 800	n = 600	n = 200
BASELINE CHARACTERISTICS AT HIV TREATMENT INITIATION^1^			
**Gender, n (%)**			
Male	275 (34.4)	206 (34.3)	69 (34.5)
Female	525 (65.6)	394 (65.7)	131 (65.5)
**Age, n (%)**			
18–30	273 (34.1)	206 (34.3)	67 (33.5)
31–40	324 (40.5)	233 (38.8)	91 (45.5)
41–50	141 (17.6)	112 (18.7)	29 (14.5)
>50	62 (7.8)	49 (8.2)	13 (6.5)
**Age, median (IQR)**	34 (28–41)	34 (29–39)	34 (28–41)
**Baseline ART Regimen, n (%)**			
3TC-TDF-EFV	663 (82.9)	506 (84.3)	157 (78.5)
3TC-TDF-NVP	62 (7.8)	33 (5.5)	29 (14.5)
Other	75 (9.4)	61 (10.2)	14 (7.0)
**Baseline CD4 Count, n (%)**			
≤100 cells/mm^3^	248 (31.0)	192 (32.0)	56 (28.0)
101–200 cells/mm^3^	263 (32.9)	202 (33.7)	61 (30.5)
201–350 cells/mm^3^	261 (32.6)	181 (30.2)	80 (40.0)
>351 cells/mm^3^	28 (3.5)	25 (4.2)	3 (1.5)
**Baseline CD4 Count, median (IQR)**	151 (70–240)	148 (61–237)	162 (87–257)

ART: antiretroviral therapy, 3TC: stavudine, TDF: tenofovir, EFV: efavirenz, NVP: nevirapine

^1^All the baseline characteristics were matched using propensity scores

### Outcomes

The primary and secondary outcomes are presented in Table 3. The majority of patients at both the HIV outpatient clinic (72%) and the PHC (82%) were reported to be alive and in care at 12 months. Being a patient at the primary health clinic appeared to be somewhat protective against death or loss to follow-up (RR: 0.66, 95% CI: 0.48–0.91). However, as S1 Fig. 1 indicates, 55 paper patient files from the original sample selected for the PHC were missing and were excluded from the primary analysis. Those patients with missing paper files (55) were identified on the electronic patient database (Tier.net); of these patients, two were excluded because they had transferred to other facilities and six were excluded because they had no baseline CD4 count. The remaining 47 were included in a sensitivity analysis, where we considered the impact of the missing files on patient outcomes at the PHC. We found that patients with missing files were more likely to be lost to follow up or dead than were those included in the main analysis. When the outcomes of the patients with missing files were included in the analysis, the difference between the sites in the proportion of patients not in care disappeared (RR: 0.91, 95% CI: 0.71–1.18). There was no difference between the sites in outcomes stratified by baseline CD4 count. We also found no difference in unsuppressed viral load or immunologic failure rates between the sites for those patients who had the necessary diagnostics reported in their files (238/800 and 205/800 for viral load and CD4 count, respectively). We thus conclude that after taking into account the effect of the missing files, the primary outcomes were comparable between the sites.

**Table 3:** Outcomes at 12 months after ART initiation with relative risk.

Outcomes	Total	HIV Outpatient Clinic	Primary Health Clinic	Relative Risk^a^
	n/N (%)	n/N (%)	n/N (%)	(95% CI)
**Primary Outcome**				
Alive and in care	595/800 (74.4)	432/600 (72.0)	163/200 (81.5)	-
Dead / LTFU	205/800 (25.6)	168/600 (28.0)	37/200 (18.5)	0.66(0.48–0.91)
≤100 cells/mm^3^	89/248 (35.9)	74/192 (38.5)	15/56 (26.8)	0.70 (0.44–1.11)
101–200 cells/mm^3^	61/262 (23.3)	45/201 (22.4)	7/61 (11.5)	0.51 (0.24–1.08)
201–350 cells/mm^3^	80/261 (30.7)	45/181 (24.9)	14/80 (17.5)	0.70 (0.41–1.21)
>351 cells/mm^3^	3/29 (10.3)	4/26 (15.4)	1/3 (33.3)	2.17 (0.35–13.6)
**Secondary Outcome**				
Unsuppressed viral load*	59/238 (24.8)	36/167 (21.6)	23/71 (32.4)	1.50 (0.96–2.34)
Immunologic failure**	10/205 (4.9)	8/139 (5.8)	2/66 (3.0)	0.53 (0.12–2.41)
SENSITIVITY ANALYSIS***				
**Primary Outcome**				
Alive and in care	739/988 (75.0)	550/741 (74.2)	189/247 (76.5)	-
Dead / LTFU	249/988 (25.0)	191/741 (25.8)	58/247 (23.5)	0.91 (0.71–1.18)

^a^Relative risk of outcome at primary health clinic, with HIV outpatient clinic as reference

* Only includes those with a viral load between 9–15 months

**Only includes those with CD4 counts between 9–15 months

***Newly matched sample including missing files

### Resource utilization and costs

Table 4 compares baseline CD4 counts, duration in care, and utilization of clinic visits and laboratory tests by site and outcome category. Not surprisingly, at both sites patients alive and in care at 12 months had a higher median baseline CD4 count than those not in care. More surprisingly, patients who did not remain in care for 12 months at the PHC spent almost twice as much time in care (7.5 months) as did patients at the HIV outpatient clinic (3.7 months). Resource utilization by site and outcome reflects these differences in duration of care. In general, resource utilization was slightly higher at the PHC than at the HIV outpatient clinic, primarily because patients at the PHC who ultimately died or were lost to follow up remained in care for an average of nearly four more months, long enough to make at least one more clinic visit and undergo an additional set of laboratory tests. For patients who did remain in care, differences between the sites were modest, with the PHC reporting slightly fewer clinic visits and slightly more laboratory tests.

**Table 4:** Time in care (months) and resource utilization for study period by outcome and facility.

	All patients		Alive in care		Dead / LTFU	
	HIV Outpatient Clinic	Primary Health Clinic	HIV Outpatient Clinic	Primary Health Clinic	HIV Outpatient Clinic	Primary Health Clinic
*Patients*, *n*	600	200	432	163	168	37
*CD4 count (cells/mm*^*3*^*)*, *median*	148	162	155	164	121	142
*CD4 count (cells/mm*^*3*^*)*, *IQR*	61–237	87–257	84–242	100–258	32–214	49–255
**RESOURCES USED IN STUDY PERIOD**						
*Average months in care*	9.7	11.2	12.0	12.0	3.7	7.5
*Number of visits*	7.0	6.6	8.7	7.3	2.7	3.5
*Laboratory tests*						
*Alanine aminotransferase*	0.6	1.0	0.8	1.0	0.2	0.9
*CD4 count*	0.5	1.7	0.7	1.9	0.1	1.1
*Creatinine*	0.7	2.2	0.8	2.4	0.3	1.2
*Full blood count*	0.6	1.0	0.8	1.0	0.2	1.0
*Viral load*	0.5	0.7	0.7	0.9	0.0	0.9
Total unit cost per visit (USD*)	31	9	31	9	31	9
Staffing cost per visit (USD*)	25	8	25	8	25	8
Fixed cost per visit (USD*)	6	1	6	1	6	1

*Costs are given in 2014 US dollars, converted at a rate of R10.83 to US$1.00

Costs by site, outcome, and cost category are presented in Table 5. Drugs and diagnostic tests are procured centrally by government and thus have identical unit costs between sites; higher costs in these categories between sites indicate higher resource usage. Patients who spend 12 months in care have similar drug and diagnostic costs between sites, indicating similar resource utilization. Unit costs for staffing and fixed costs are specific to the site, reflecting staffing composition, infrastructure, equipment, and patient volume. Differences in these cost categories can thus reflect differences in unit costs and/or resource usage.

**Table 5:** Mean 12 month cost per patient (USD) by outcome and facility, broken down by cost category.

	All patients (USD)		Alive in care (USD)		Dead / LTFU (USD)	
*Cost category*^1^ *(mean*, *%)*	HIV Outpatient Clinic	Primary Health Clinic	HIV Outpatient Clinic	Primary Health Clinic	HIV Outpatient Clinic	Primary Health Clinic
*Drugs*	85 (25%)	100 (47%)	108 (25%)	113 (47%)	28 (23%)	43 (43%)
*Diagnostics*	41 (12%)	51 (24%)	53 (12%)	57 (24%)	11 (9%)	24 (24%)
*Staffing*	177 (52%)	56 (26%)	219 (51%)	62 (26%)	68 (56%)	30 (30%)
*Fixed*	39 (11%)	6 (3%)	48 (11%)	7 (3%)	15 (12%)	3 (3%)
***Average cost per patient (95% CI)***	342 (326, 358)	213 (204, 222)	428 (422, 452)	238 (233, 244)	122 (106, 137)	100 (87, 113)

^1^Costs are given in 2014 US dollars, converted at a rate of R10.83 to US$1.00

The average cost per patient initiated on ART over the 12 months following initiation was significantly lower at the PHC ($213) than at the HIV outpatient clinic ($342). This finding of lower costs at the PHC held for all outcomes and remained valid even when the longer duration of care for those ultimately not in care was taken into account: $20 v. $36 per month at the PHC and HIV outpatient clinic, respectively, for patients in care and $13 v. $33 per month for patients not in care. The cost per month in care was calculated by dividing the total average cost per patient (Table 5) by the average months in care (Table 4). Most of this difference derives from the higher costs of staff and infrastructure at the HIV outpatient clinic. While patients at the HIV outpatient clinic made slightly more visits, the cost per visit was substantially more ($31 v. $9).

## Discussion

In this analysis of outcomes and costs of providing antiretroviral therapy under South Africa’s two major models of service delivery, we found that both models achieved similar proportions of patients alive and in care 12 months after initiation, but the primary health clinic model cost substantially less per patient treated, despite providing more months of patient care overall. This cost difference is due largely to the higher staff and infrastructure costs incurred in a hospital-based clinic, and reflect the fact that ART patients rarely require the more specialized services and equipment available at a hospital.

Other studies in South Africa have consistently found that decentralization to PHCs from centralized clinics and task shifting to nurses produce favorable or equivalent results for patients [14–16, 20, 21, 24]. We previously showed that down-referral of stable ART patients to PHCs after initiation at a hospital-based HIV outpatient clinic reduced the costs of managing stable patient [17]. The study reported here, which demonstrated both equivalent patient outcomes and lower costs for PHC-level, nurse-led initiation and management, stands in contrast to the only other cost estimate of this model we discovered, from the STRETCH trial in South Africa’s Free State Province. The STRETCH trial found that nurse initiation and management can improve health outcomes and quality of care somewhat, but at a higher mean cost [24, 25] that reflected high setup and implementation costs and more clinic visits per patient in the nurse-led arm. Patients in the STRETCH trial were enrolled prior to the advent of NIMART and were managed under the constraints of a randomized controlled trial. Our study is therefore more likely to reflect current, actual conditions in the public sector since NIMART became the standard of care. The knowledge that PHCs can deliver equally good care, at much lower cost, will help the South African Government, neighboring governments, and their partners to make cost-effective decisions about program design.

Shifting ART responsibilities from doctors to nurses has occurred to varying degrees across all treatment facilities in South Africa. At PHCs, nurses provide nearly all HIV services, with only occasional referral, and HIV care is largely integrated with other primary health care. HIV outpatient clinics at hospitals remain stand-alone services with relatively significant doctor involvement, with NIMART trained nurses working alongside but not always replacing doctors. We found that this staffing mix, which also affects patient volume and the efficiency of staff time use, is one of the two main causes of the high cost of HIV outpatient clinics (the other is simply the higher cost of hospital infrastructure). The importance of the staff complement suggests that a re-allocation of responsibilities, with complicated patients (e.g. patients with very low CD4 counts at initiation, patients failing first line therapy, patients on second line therapy) referred to hospital-based HIV clinics and the vast majority of uncomplicated patients initiated and managed at PHCs. Supporting this recommendation, a study of the NIMART rollout in the City of Johannesburg found that NIMART increased ART initiations at PHCs and reduced them at hospital-based HIV clinics, allowing the hospital clinics to focus on more difficult cases [22].

Although we estimated both costs and effectiveness of the two models of ART delivery, our study was not designed as a cost-effectiveness analysis (CEA), as we do not believe that a choice can be made between the two models. In view of the vast demand for HIV treatment in South Africa, both models will continue to be required for years to come. The information we report will help support efficient allocation of patients between facilities, decisions about future expansion of treatment capacity, and planning and budgeting.

This study had a number of limitations, most of which are common to retrospective, observational cohort studies. Most important, the study was limited to a single pair of sites in one province, which limits geographic generalizability. The study sites are, however, public sector treatment facilities following the national treatment guidelines, and are thus likely to be typical of the public sector in other provinces in South Africa, if not of other countries. Although we matched our samples carefully using a rigorous matching algorithm, moreover, unobserved differences between the samples may bias our estimates. With small samples and limited covariates its possible that although individual patients were matched on their p-score some residual confounding may remain. Our results are dependent on the accuracy and completeness of routinely collected patient records, which may also have varied by site. Finally, despite our access to relatively recent data, the rapid pace of change of national and global treatment guidelines makes it nearly impossible for research to “keep up” with the current treatment landscape.

In spite of these limitations, we conclude that initiating and managing ART patients at primary health clinics under South Africa’s NIMART policy is producing equally good outcomes as hospital-based HIV outpatient clinic care at much lower cost. Evolution of the country’s hospital-based clinics into well-resourced, centralized, expert referral facilities that focus on complicated patients, while investing program expansion resources into PHCs, may thus be the country’s preferred strategy. There are currently no widely-utilized guidelines for how to triage patients between routine PHC care and specialized HIV clinic care. Developing guidance in this area may thus be a priority for policy makers.

While our findings should support the continued expansion of NIMART, we conclude by noting that the impact of the program on non-HIV care must also be considered. So far, nurses appear to have incorporated HIV care into their workloads smoothly, but there is no evidence to suggest how much additional capacity remains on the system or that the addition of HIV care has not been to the detriment of other patients. While we have looked specifically at the provider costs of ART, a full evaluation of the NIMART program—and of similar decentralizing and task-shifting initiatives in other countries—should take into account both the impact of the program on non-HIV care and its benefits and costs to patients themselves.

## Supporting Information

S1 Fig Study Samples. (PDF)
