# Supplementary figures and images for: Treatment Outcomes and Costs of Providing Antiretroviral Therapy at a Primary Health Clinic versus a Hospital-Based HIV Clinic in South Africa
Source: PLoS One. 2016 Dec 12;11(12):e0168118. doi: 10.1371/journal.pone.0168118 (PMC5152901; doi:10.1371/journal.pone.0168118)

## PHC FACILITY

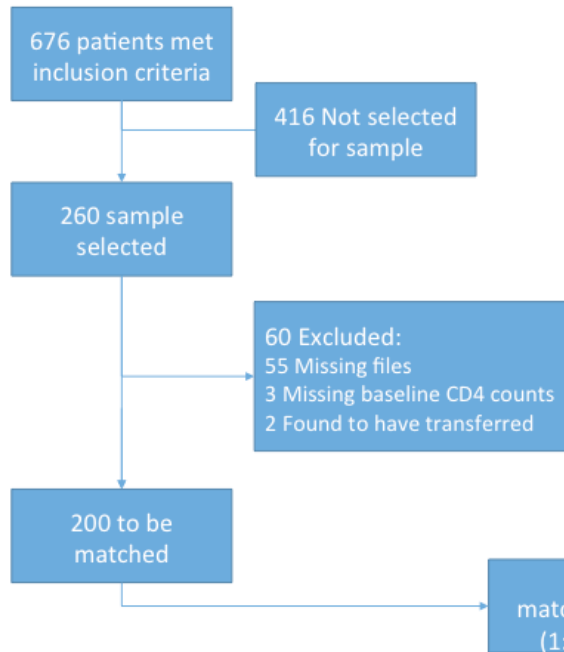

## HIV OUTPATIENT FACILITY

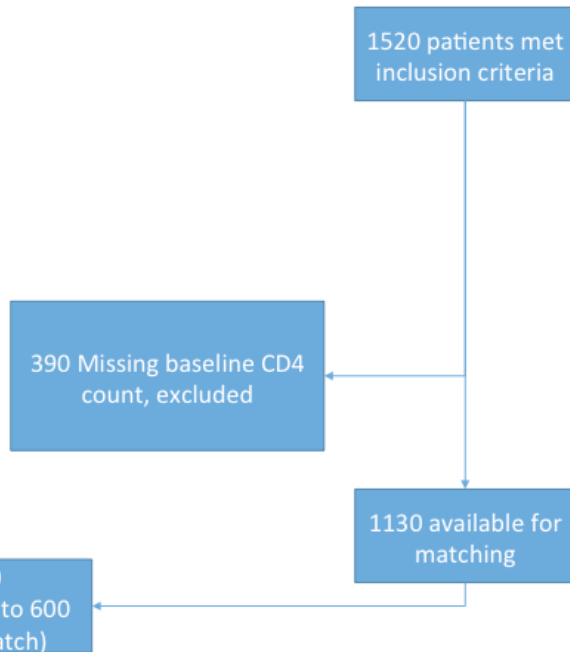

Supplement: S1 Fig — (PDF) [file pone.0168118.s001.pdf]
